# Effectiveness of Booster Vaccinations on the Control of COVID-19 during the Spread of Omicron Variant in Malaysia

**DOI:** 10.3390/ijerph20021647

**Published:** 2023-01-16

**Authors:** Matthew Tze Jian Wong, Satvinder Singh Dhaliwal, Venugopal Balakrishnan, Fazlina Nordin, Mohd Nor Norazmi, Gee Jun Tye

**Affiliations:** 1 Institute for Research in Molecular Medicine (INFORMM), Universiti Sains Malaysia, Minden 11800, Malaysia; 2 Curtin Health Innovation Research Institute, Faculty of Health Sciences, Curtin University, Perth 6102, Australia; 3 Duke-NUS Medical School, National University of Singapore, Singapore 119077, Singapore; 4 Office of the Provost, Singapore University of Social Sciences, Singapore 599494, Singapore; 5 Centre for Tissue Engineering and Regenerative Medicine (CTERM), Faculty of Medicine, Universiti Kebangsaan Malaysia, Kuala Lumpur 56000, Malaysia; 6 School of Health Sciences, Universiti Sains Malaysia, Kubang Kerian 16150, Malaysia; 7 Malaysian Genome and Vaccine Institute, National Institutes of Biotechnology Malaysia, Kajang 43000, Malaysia

**Keywords:** COVID-19, Omicron, booster, vaccine effectiveness, Malaysia

## Abstract

(1) Background: The assessment of vaccine effectiveness against the Omicron variant is vital in the fight against COVID-19, but research on booster vaccine efficacy using nationwide data was lacking at the time of writing. This study investigates the effectiveness of booster doses on the Omicron wave in Malaysia against COVID-19 infections and deaths; (2) Methods: This study uses nationally representative data on COVID-19 from 1 January to 31 March 2022, when the Omicron variant was predominant in Malaysia. Daily new infections, deaths, ICU utilization and Rt values were compared. A screening method was used to predict the vaccine effectiveness against COVID-19 infections, whereas logistic regression was used to estimate vaccine effectiveness against COVID-19-related deaths, with efficacy comparison between AZD1222, BNT162b2 and CoronaVac; (3) Results: Malaysia’s Omicron wave started at the end of January 2022, peaking on 5 March 2022. At the time of writing, statistics for daily new deaths, ICU utilization, and effective reproductive values (Rt) were showing a downtrend. Boosted vaccination is 95.4% (95% CI: 95.4, 95.4) effective in curbing COVID-19 infection, compared to non-boosted vaccination, which is 87.2% (95% CI: 87.2, 87.2). For symptomatic infection, boosted vaccination is 97.4% (95% CI: 97.4, 97.4) effective, and a non-boosted vaccination is 90.9% (95% CI: 90.9, 90.9). Against COVID-19-related death, boosted vaccination yields a vaccine effectiveness (VE) of 91.7 (95% CI: 90.6, 92.7) and full vaccination yields a VE of 65.7% (95% CI: 61.9, 69.1). Looking into the different vaccines as boosters, AZD1222 is 95.2% (CI 95%: 92.7, 96.8) effective, BNT162b2 is 91.8% (CI 95%: 90.7, 92.8) effective and CoronaVac is 88.8% (CI 95%: 84.9, 91.7) effective against COVID-19 deaths. (4) Conclusions: Boosters are effective in increasing protection against COVID-19, including the Omicron variant. Given that the VE observed was lower, CoronaVac recipients are encouraged to take boosters due to its lower VE.

## 1. Introduction

COVID-19 is a term familiar to almost everyone today. For the past few months, there has been a massive surge in cases, and along with it, fears regarding the Omicron variant of COVID-19. The B.1.1.529 COVID-19 variant was initially detected in South Africa, first reported to the World Health Organization (WHO) on 24 November, identified from a specimen collected on 9 November 2021. However, due to a quick rise in cases of this variant in South Africa, it was classified as a variant of concern (VOC) with the name Omicron by the World Health Organization Technical Advisory Group on SARS-CoV-2 Virus Evolution (WHO TAG-VE) on 26 November 2021. Currently, the most common sub-lineages of Omicron are BA.1, BA.1.1, and BA.2 [1].

The rise of the Omicron variant remains a topic of great concern among scientists worldwide, as it harbours mutations that have potential implications on vaccine effectiveness, viral transmissibility, disease severity and immune evasion [2,3]. As of 29 December 2021, 37 mutations on the spike or S protein were identified, with some being found in other VOCs such as the alpha, beta, gamma and delta variant [4]. The primary fear is the mutations associated with the spike protein’s receptor-binding domain (RBD) that facilitates binding with the angiotensin-converting enzyme 2 (ACE2) for viral entry into host cells [5]. Research has shown that the mutations on the RBD of Omicron have caused stronger binding between RBD and ACE2 [6]. These findings are worrying as most COVID-19 vaccines in the current market induce humoral immunity that identifies the spike protein as the target [7]. Therefore, the mutations, particularly in the RBD region, are worrying because they can reduce the efficacy of current vaccination regimens, and thus cause a colossal setback in global efforts to make COVID-19 endemic. 

Malaysia’s first Omicron case was reported on 3 December 2021, where the patient was a traveller from South Africa, the origin of the Omicron variant. Since then, the Omicron variant has been rampant across the country, surpassing the transmission of the previous majority Delta variant. From the genomic surveillance of COVID-19 in Malaysia, in December 2021, about 50% of the samples processed were of the Omicron variant, with all of them being imported cases detected upon international arrival. However, in January 2022, the percentage of Omicron cases increased to approximately 70%, with most of the cases being imported but some starting to appear in multiple states across the country. In February 2022, 98.7% of GISAID submissions by Malaysia were of the Omicron variant [4,8]. In order to handle the COVID-19 pandemic in Malaysia, Act 342—Prevention and Control of Infectious Diseases Act 1988 was enforced [9]. Under this enforcement, as listed in the COVID-19 guidelines issued by the Ministry of Health Malaysia [10], all suspected cases, probable cases, and close contacts must be tested using either an antigen rapid test kit (RTK-Ag) or reverse-transcription polymerase chain reaction (RT-PCR). It is mandatory by law for individuals with positive test results to report to the ministry. For close contacts, they are issued a home surveillance order to undergo compulsory home isolation and report antigen rapid test kit results during the quarantine period. Likewise, vaccination against COVID-19 is a nationwide government initiative, and all vaccination and verification is carried out through a single nationwide system known as MySejahtera, even if the vaccine is administered in private healthcare facilities or outstations. Official COVID-19 data is updated and released to the public on a daily basis through the COVIDNow website, which is sourced from a GitHub public repository maintained by the Ministry of Health Malaysia.

To overcome the waning of vaccine effectiveness caused by the Delta variant, the Malaysian government decided to introduce the National COVID-19 Immunization Programme—Booster (PICK-B) on 13 October 2021. At the start, the program prioritizes the administration of booster doses to high-risk individuals and frontliners [11]. In addition, recipients of CoronaVac, as the primary vaccination regimen, were included in the priority list due to the observation that numerous studies showed that CoronaVac has lower efficacy than other brands, providing limited protection against COVID-19 VOCs [12]. After that, booster doses were administered to all fully vaccinated individuals, involving the cooperation between both the government and the private health sector from 30 September 2021 onwards [11]. The top three types of vaccines administered are BNT162b2 (Pfizer-BioNTech Comirnaty), CoronaVac (Sinovac) and AZD1222 or ChAdOx1 (Oxford-AstraZeneca Vaxzevria) [13], administered to 41.4%, 30.7% and 6.2% of the population, respectively, as of 31 March 2022.

Currently, most data on the vaccine effectiveness of COVID-19 vaccines have been gathered mainly through randomized controlled trials (RCTs). It is indeed understandable why information obtained from RCTs is generally preferred by many researchers, due to its strict inclusion criteria, continual observation and specific populations, which ensures high internal validity [14]. However, the stringent controlled settings of RCTs do not represent the real-world effectiveness of the vaccines. Real-world data provide valuable information outside the scope of RCTs, such as any unpredicted outcomes in the real-world pertinence of antibody responses after vaccination. The study of real-world effectiveness also takes into account factors such as the rollout and distribution of vaccines, vaccine supply and demand dynamics and government policies [15]. These elements are particularly relevant in low- and middle-income countries (LMICs) in the course of mitigating COVID-19 fatalities in respective countries via vaccination [16].

Therefore, this study aims to investigate the effects of booster doses on the Omicron wave in Malaysia by using real-world data established from nationally representative statistics on COVID-19 cases, deaths and vaccinations, and thereby estimating vaccine effectiveness against COVID-19 infections and deaths, comparing a full vaccination and a boosted vaccination regimen. A comparison will also be made between the major three vaccine brands authorized for use in Malaysia.

## 2. Materials and Methods

### 2.1. Data Sources

In Malaysia, it is compulsory for all medical institutions to report COVID-19 cases and deaths to the Ministry of Health Malaysia, as required under the Act 342—Prevention and Control of Infectious Diseases Act 1988. Thus, all COVID-19-related data, both granulated and aggregated, including vaccinations, cases and deaths, are published in the Ministry of Health’s COVID-19 Data Repository on GitHub as part of the national surveillance efforts on the COVID-19 pandemic in Malaysia [13], and are open-access. However, COVID-19-related intensive care unit (ICU) admissions individual-level data are not published in the repository. Because of this, only COVID-19 related death will be used as the indicator of a severe outcome of the disease. The estimate of the current population of Malaysia for the year 2021 was obtained from the repository, which in turn was sourced from the Department of Statistics Malaysia. Furthermore, daily effective reproduction values (Rt) of COVID-19 were obtained from the daily press statement on the pandemic situation by the Director-General of Health Malaysia, Datuk Dr. Noor Hisham Abdullah [17]. Two methods will be used in this study to analyse the data. The screening method will be used first to ascertain the VE against COVID-19 infection and COVID-19 symptomatic infection. On the other hand, multivariable logistic regression will be used to calculate the VE against COVID-19 deaths.

### 2.2. Study Design, Group and Methodology of the Determination of Vaccine Effectiveness against COVID-19 Infection

The screening method used in this study to measure vaccine effectiveness is adapted from Orenstein et al. [18]. Data on the national level were obtained, including all individuals who were fully vaccinated (received two doses for a two-dose vaccine regimen and one dose for a one-dose vaccine regimen) and boosted (received three doses for two-dose vaccines and received at least two doses for one-dose vaccines) against COVID-19, as of 31 March 2022. Moreover, positive COVID-19 cases were chosen from 1 January 2022 to 31 March 2022, as the Omicron variant was prevalent in Malaysia after January 2022, with 98.7% of cases being of the Omicron variant by February 2022 [4]. Vaccine effectiveness (VE) with 95% confidence intervals is calculated using the equation below:

(1) $$VE_{Y,i}=\frac{PPV_{d}-PCV_{Y,d}}{PPV_{d}\times \left(1-PCV_{Y,d}\right)}$$

This screening technique predicts the vaccine effectiveness against an outcome of a disease *Y* (total infection and specifically, symptomatic infection of COVID-19), for the vaccination status *d* (full or boosted vaccination, encompassing all types of vaccine), using the following variables calculated collectively:

- The proportion of the population that is vaccinated, PPV;
- The proportion of outcomes within the vaccinated population, PCV.

Further details on this method can be found in the Appendix A.

Vaccine effectiveness against two outcomes of infections was determined using this method, namely against total confirmed COVID-19 infections and symptomatic confirmed COVID-19 infections. The case definitions for each outcome are listed in Table 1.

### 2.3. Study Design, Group and Methodology of the Determination of Vaccine Effectiveness against COVID-19 Deaths

The study design employed in determining vaccine effectiveness against COVID-19 deaths is a retrospective cohort study. The study population (*n* = 1,158,235) comprised all RTK-Ag or RT-PCR-confirmed positive COVID-19 cases, including deaths, aged 18 and above within the three-month period from 1 January 2022 to 31 March 2022. These criteria represent those under the PICK-B program eligible to receive a booster dose and the period where the Omicron variant was prevalent. Individuals who received a heterologous primary vaccination regimen (excluding a booster dose) or had unverified vaccination records were excluded. Only fully vaccinated individuals who received homologous BNT162b2, CoronaVac, and the AZD1222 primary vaccination schedule (≥14 days post-administration of the second dose) were included, as they represent the top three vaccine brands administered in Malaysia. An individual is considered boosted when a booster dose of the above vaccine types was administered (≥12 days post-administration of the third dose). Twelve days after receipt of the booster dose was chosen as the cut-off threshold, as it was proven that immunologic protective effects from boosters could be seen after this period [19].

Vaccination status, primary vaccination regimen type and booster vaccination type were used to predict deaths by COVID-19 within the same time period using multivariable logistic regression, after adjusting for covariates. This approach is similar to the study of vaccine effectiveness among Malaysians conducted by Suah et al. [20]. Covariates included in the model include (i) age, (ii) age-squared, (iii) gender, (iv) nationality, (v) state and (vi) the presence of comorbidities. Age was included in the model as a continuous variable. Age-squared allowed for non-linearity in the influence of age on the probability of death by COVID-19. This approach was employed due to the absence of individual-level data on healthy individuals that were uninfected by COVID-19.

Statistical analyses were conducted using Microsoft Excel 365 (Version 2201) and IBM SPSS Statistics (Version 28).

## 3. Results

### 3.1. Daily New Infection, New Deaths, ICU Utilization and Rt Values

**Figure 1:**
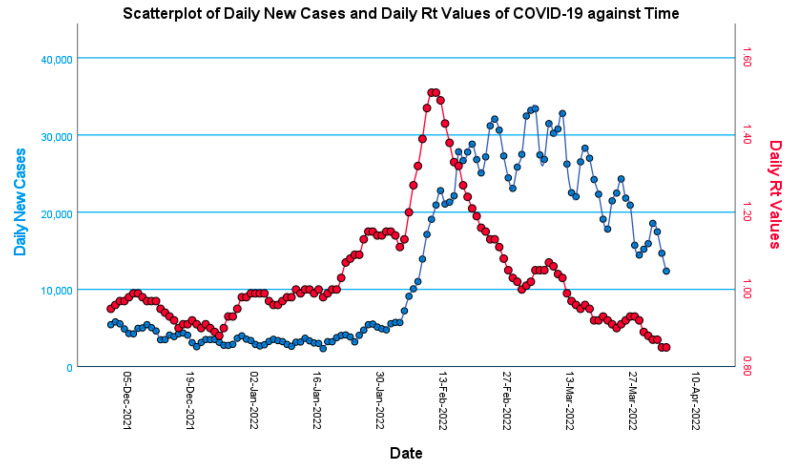
Scatter plot of daily Rt values (red line) and daily new cases of COVID-19 (blue line) in Malaysia against time, from 1 January 2022 to 31 March 2022. Raw data were sourced from GitHub repository by Ministry of Health Malaysia [13].

**Figure 2:**
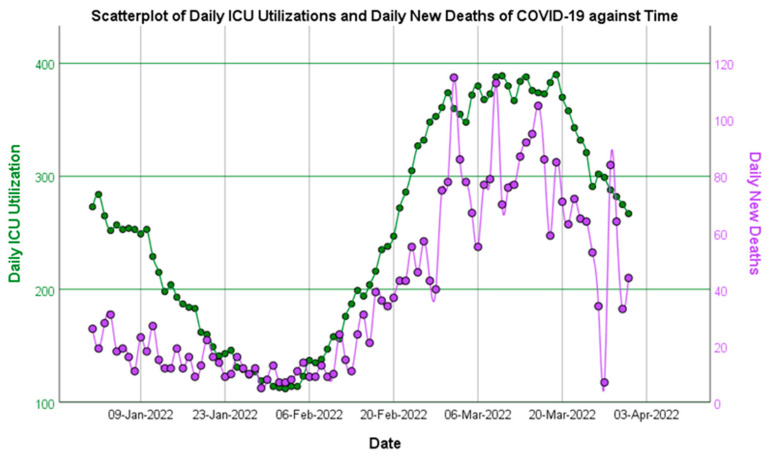
Scatter plot of daily ICU utilization (green line) and daily new deaths (purple line) of COVID-19 in Malaysia against time, from 1 January 2022 to 31 March 2022. Raw data were sourced from GitHub repository by Ministry of Health Malaysia [13].

Looking at the daily new cases of COVID-19 in Malaysia in Figure 1, an epidemic wave of the Omicron variant could be seen clearly, with its starting point at the end of January 2022 and its peak on 5 March 2022, with 33,406 new cases. After that, daily new cases dropped, starting the downward trend of the Omicron wave. Daily Rt values also showed a similar trend but preceded the daily new cases, with a more prominent wave shape where its peak was on 10 and 11 February 2022, with the estimated Rt value of 1.51 for both days. As for the daily ICU utilization and new death cases, as seen in Figure 2, the general trends between both variables are similar, with the rise of daily ICU utilization and new deaths during the Omicron wave, albeit the daily new-deaths data had a much higher fluctuation. Towards the end of March, both variables have shown a downwards trend.

### 3.2. Booster Vaccine Effectiveness against COVID-19 Infection

Overall, looking at the vaccine effectiveness predicted using the screening method, receiving a booster dose is more effective in curbing COVID-19 infection. As seen in Table 2, the screening method predicts that full vaccination is 87.2% (95% CI: 87.2, 87.2) effective and boosted vaccination is 95.4% (95% CI: 95.4, 95.4) effective against COVID-19 infection. For symptomatic COVID-19 infection, full vaccination is 90.9% (95% CI: 90.9, 90.9) effective and boosted vaccination is 97.4% (95% CI: 97.4, 97.4) effective against it. Variables that were used to calculate the VE against COVID-19 infection and age-stratified estimation of VE against COVID-19 infection can be found in Table A1 and Table A2, respectively.

### 3.3. Booster Vaccine Effectiveness against COVID-19 Deaths

The characteristics of the study cohort are listed in Table A3. Vaccine effectiveness in preventing deaths among confirmed COVID-19 cases according to primary and booster vaccine type and vaccination status is listed in Table 3. In general, it shows that booster vaccination is more effective in preventing death among COVID-19 patients compared to full vaccination, where booster vaccination yields a VE of 91.7 (95% CI: 90.6, 92.7) and full vaccination yields a VE of 65.7% (95% CI: 61.9, 69.1), regardless of primary and booster vaccine types. Unfortunately, the VE of CoronaVac booster with either BNT162b2 or AZD1222 primary cannot be calculated as there were insufficient data. Among the three vaccine types, AZD1222 was most effective, followed by BNT162b2 and subsequently CoronaVac. As for booster vaccination, regardless of the primary vaccination type, receiving a third dose of AZD1222 showed a higher VE (95.2%, CI 95%: 92.7, 96.8) than BNT162b2 (91.8%, CI 95%: 90.7, 92.8), and CoronaVac (88.8%, CI 95%: 84.9, 91.7). A similar trend was observed when primary regimens were stratified into the different vaccine types. 

## 4. Discussion

Marked by daily new infections, new deaths, ICU utilization, and Rt values of COVID-19, the Omicron wave commenced in January 2022, peaking in the first half of March 2022, and then showed a downwards trend. Such a trend signifies the probable success of booster vaccination in curbing the Omicron wave in Malaysia, as booster vaccination rates steadily increased, peaking in mid-January, with an average of over 200,000 new booster vaccines being administered daily [13]. Although the wave had not officially ended at the time of writing, it was postulated that the downtrend would continue as the wave had already peaked, and ICU utilization and deaths were trending downwards as well. In addition, Rt values also indicated that the downtrend would continue, possibly towards the end of the Omicron wave. One of the potential factors that contributed to the downtrend was the achievement of herd immunity within the Malaysian population, through a high quantity of infections and the partial protection conferred by primary and boosted vaccinations. It is noteworthy that Malaysia’s Rt values were much lower compared to other countries [21], due to Malaysia being a tropical country and not a four-season country. Hence, the warm weather all year round might have prevented the worsening of COVID-19.

This study evaluates the importance of the booster vaccine rollout to Malaysians in curbing the COVID-19 pandemic. Generally, the results from this study showed that boosted vaccination is more effective in preventing total and symptomatic COVID-19 infections during the Omicron wave in Malaysia than those with two-dose vaccination without a booster. The result of this study is consistent with other studies on the effectiveness of booster doses against VOCs [22]. Unfortunately, vaccine effectiveness against COVID-19 infections could not be stratified by vaccine types as there is an absence of individual-level data on uninfected individuals, and the chosen screening method does not allow such stratification. Hence, vaccine effectiveness between different vaccines can only be postulated from the results of vaccine effectiveness against COVID-19 deaths that provides for such stratification. As for deaths among COVID-19 patients, the same trend could be observed where boosted vaccination is more effective than without a booster. Research has shown that boosters reactivate memory B cells against the spike protein, which were capable of recognizing the mutated Omicron RBD. Boosters also enhance T-cell reactivity towards Omicron [23,24].

The waning of vaccine effectiveness for two doses was expected due to the Omicron variant. Suah et al. reported that during the Delta wave in Malaysia, the efficacy of CoronaVac and BNT162b2 was reduced compared to the previous waves of COVID-19 [20]. Nevertheless, COVID-19 booster vaccines of any kind were proven effective in lowering COVID-19 infections and deaths in Malaysia. Although two doses were still providing some degree of protection at the time of writing, it was still considered vital and highly encouraged for boosters to be taken to confer a higher degree of protection against COVID-19 including the Omicron variant, especially for CoronaVac recipients and senior citizens. 

Looking at the VE against deaths, AZD1222 and BNT162b2 showed better protection against COVID-19, followed by CoronaVac. Despite this, the protection the two vaccines elicited without a booster against death was 75.1 (95% CI: 68.0, 80.6) and 72.3 (95% CI: 68.9, 75.3), respectively. VE could not be calculated when CoronaVac was taken as a booster for AZD1222 and BNT162b2 primary schedules. This is because under the PICK-B program, the recipients of both primary schedules were recommended to take either AZD1222 or BNT162b2 as boosters. Therefore, there were too few individuals who had taken CoronaVac as the booster to calculate the VE accurately. AZD1222 is an adenovirus vector-based vaccine encoding the full-length spike protein sequence, whereas BNT162b2 is an mRNA-based vaccine encoding the RBD of the S1 protein only [25].

Various articles and preprints indicated that two doses of AZD1222 or BNT162b2 were insufficient to protect against the Omicron variant, and boosting was required to reinstate significant protection [26,27]. Our study has shown that a booster of any kind increased the VE against death to 95.1 (95% CI: 92.9, 96.6) and 93.3 (95% CI: 92.1, 94.3) for AZD1222 and BNT162b2 primary recipients, respectively. This result is in line with other studies. For instance, an AZD1222 booster increased neutralizing antibody titres by 3.25-fold and 5.33-fold for AZD1222 and BNT162b2 primary recipients, respectively, in a phase II trial conducted in the United Kingdom [28]. Furthermore, a Danish study (preprint) revealed that the VE of BNT162b2 primary vaccination was only 55.2% (95% CI: 23.5, 73.7) against the Omicron variant, but the VE dropped significantly to 9.8% after five months. A homologous booster administered within five months post-second dose restored the VE to 54.6% (95% CI: 30.4, 70.4) [29]. Moreover, in this study, it is interesting to note that taking BNT162b2 as a heterologous booster for AZD1222 primary recipients showed better VE compared to a homologous booster. It was proven that a heterologous prime-boost vaccination of AZD1222-BNT162b2 is more effective than BNT162b2-AZD1222. Nevertheless, for BNT162b2 primary recipients, a homologous prime-boost was more effective than heterologous prime-boost vaccinations [30].

For CoronaVac, the primary two-dose regimen was marginally effective to confer protection in this study. A booster dose of any brand is needed to prevent deaths with 89% efficacy, presumably preventing COVID-19 infections. Research has shown that the RNA-based and viral vector-based vaccines generate a more robust immune response than their inactivated virus-based counterparts. The vaccine candidate for CoronaVac was the inactivated virus of the early outbreak CN2 strain from China [31]. Clinical trials have also shown waning vaccine efficacy for CoronaVac towards VOCs, going down to a VE of 50.7% [32]. Nevertheless, CoronaVac still has advantages. Antibodies elicited by BNT162b2 can only bind to the spike protein, while CoronaVac-generated antibodies can bind to both the spike protein and the nucleocapsid [33]. Furthermore, CoronaVac is superior in stimulating CD4^+^ and CD8^+^ T-cell responses to the structural protein than BNT162b2, potentially compensating for the waning of neutralizing antibody protection [34]. At the time of writing, we are currently determining whether CoronaVac boosters to primary vaccination with BNT162b2 or AZD1222 would stimulate better T cell responses against the spike protein than boosters of the other two vaccine types. Nevertheless, it should be noted that VE from other studies is not to be used for direct comparison of VE in our study, as the methods of analysis used and the sample sizes are vastly different. The VE from other studies is cited here to provide evidence regarding the waning of vaccine effectiveness over time and a crude comparison of VE between the three vaccine brands.

Data presented here have shown that it is recommended that CoronaVac recipients be boosted by BNT162b2 and AZD1222. Furthermore, many studies have supported heterologous prime-boost vaccination for CoronaVac recipients as it has been shown to improve the immunological responses generated. For instance, a study in Turkey showed that a heterologous booster of BNT162b2 given to CoronaVac recipients resulted in a 104.8-fold increase in antibody levels. In contrast, a homologous CoronaVac booster caused an 8.7-fold rise [35]. Another study demonstrated that for CoronaVac recipients, boosters have resulted in a geometric fold-increase of IgG titre of 152 (95% CI: 134, 173) for BNT162b2, 90 (95% CI: 77, 104) for AZD1222, and 12 (95% CI: 11, 14) for CoronaVac. This information benefits older populations with weaker immune responses to elicit better protection against COVID-19 [36].

The strength of this study is being one of the first to determine the efficacy of a booster vaccine against the Omicron variant using nationwide data instead of smaller-scale clinical trials. In addition, data on COVID-19 in Malaysia used in this study were obtained from a firmly established official and direct repository under the Ministry of Health Malaysia. Because of this, the results from this study will contribute greatly to real-world data on booster vaccine effectiveness. This study also demonstrates the effects of booster vaccinations in LMICs. However, this study also contains some limitations. First, using death as an indicator for the severe outcome of COVID-19 is insufficient in evaluating vaccine effectiveness. However, based on WHO guidelines [37], using deaths as severe outcome is plausible in this case as our nationwide database in terms of COVID-19 infections, hospitalization and vaccination is accurate and up-to-date, as it is mandatory by law. Using this, we demonstrated a large vaccine impact on COVID-19. Still, the method of analysis in this study was dependent on the granularity of data. Aggregated data on COVID-19-related ICU admissions could not be used in the screening method as a severe outcome due to the lack of data stratified into the vaccination status, i.e., unvaccinated, fully vaccinated and boosted. Therefore, VE against infection can only be estimated, using a combination of mild and severe outcomes using screening method. Moreover, as the data on the type of vaccine received were only available for cases (infected) but not controls (uninfected), the VE against infection could only be estimated using the screening method, not multivariable logistic regression. Had it been possible, the same method would have been carried out for both analyses. Furthermore, at the time of writing, genomic surveillance of COVID-19 was still inadequate in Malaysia, as the submission of sequenced data to GISAID was voluntary. Hence, variant-specific vaccine effectiveness, including sub-lineages such as Omicron BA.1, BA.1.1 and BA.2, could not be determined. The spreading of the Omicron variant could only be postulated from the GISAID data trend and the media statement of the Ministry of Health Malaysia. 

At the time of writing, the Ministry of Health Malaysia had ruled that all non-boosted CoronaVac and Sinopharm BBIBP primary vaccine recipients above 18 years old and all non-boosted senior citizens above 60 years old, regardless of primary vaccination, had lost their status as fully vaccinated. This move reinforced the urgency of getting a booster dose to enhance antibody levels, as CoronaVac’s efficacy had been shown to drop substantially over time, and senior citizens are high-risk individuals [38]. After 1 April 2022, Malaysia officially entered the “Transition to Endemic” phase since all indicators such as cases, ICU admissions, and death were on a downward trend. As a result, national border restrictions were lifted, and more social activities were allowed with relaxed standard operating procedures compared to previous phases. Although the peak of COVID-19 cases was higher, COVID-19-related ICU admissions and deaths were, at the time of writing, 80–90% lower during the Omicron wave than during the Delta wave [39]. This accomplishment can be attributed to the success of the PICK-B program in distributing and administrating boosters, thereby bringing down the cases to a controllable level, as proven by this study. However, at the time of writing, mask-wearing was mandatory and social distancing measures were highly encouraged to continue the promising downward trend of COVID-19 cases.

## 5. Conclusions

The results of our study could serve as a guide to policy decisions in LMICs in terms of the rollout of booster doses. It was also recommended that the monitoring of vaccine efficacy over time is continued to ascertain the need for further booster doses. Furthermore, efforts on genomic surveillance should be strengthened to help researchers investigate variant-specific vaccine efficacy, notably when new variants like XE are still appearing, posing the potential threat of shifting COVID-19 away from heading towards the endemic phase. However, as for the Omicron wave, our findings indicate the success of a third booster dose in curbing COVID-19 in Malaysia, at least at the time of writing. 

## Figures and Tables

**Table 1:** COVID-19 case definitions [10].

Case Definition	Criterion
Confirmed COVID-19 Infection	A person with a positive Nucleic Acid Amplification Test (NAAT); RT-PCR, Rapid Molecular, and Gene X-pert
	OR
	A person with a positive SARS-CoV-2 RTK-Ag
	OR
	An asymptomatic person with a positive SARS-CoV-2 RTK-Ag AND who is a contact of a probable or confirmed case
Symptomatic COVID-19 Infection	Any confirmed COVID-19 infection with the following criterion: 1. Acute onset of fever AND cough
	OR
	2. Acute onset of ANY TWO OR MORE of the following signs or symptoms: fever, cough, general weakness/fatigue, headache, myalgia, sore throat, coryza, dyspnoea, anorexia/nausea/vomiting, diarrhoea, altered mental status
	OR
	3. Recent onset of anosmia (loss of smell) or ageusia (loss of taste) in the absence of any other identified cause

**Table 2:** Proportion of COVID-19 cases within the vaccinated population, the proportion of the population vaccinated against COVID-19 and vaccine effectiveness estimates according to vaccination status and presence of symptoms during infection.

	PCV (%)	PPV (%)	VE (%), 95% CI
**COVID-19 Infection**			
Vaccinated at least two doses	4.6	79.0	91.5 (91.5, 91.5) *
• Full Vaccination	5.3	30.6	87.2 (87.2, 87.2)
• Boosted Vaccination	4.1	48.4	95.4 (95.4, 95.4)
**Symptomatic COVID-19 Infection**			
Vaccinated at least two doses	3.0	79.0	94.2 (94.2, 94.2) *
• Full Vaccination	3.9	30.6	90.9 (90.9, 90.9)
• Boosted Vaccination	2.4	48.4	97.4 (97.4, 97.4)

* Weighted sum of full and boosted vaccination VE. Abbreviations: PCV, proportion of cases in vaccinated population; PPV, proportion of population vaccinated; VE, vaccine effectiveness; CI, confidence intervals.

**Table 3:** Vaccine effectiveness estimates in preventing deaths among COVID-19 cases, according to vaccination status, types of vaccines for primary regimen and booster.

	VE (%), 95% CI			
	Primary Vaccination Regimen			
	All Types	AZD1222	BNT162b2	CoronaVac
**Fully Vaccinated**	65.7 (61.9, 69.1)	75.1 (68.0, 80.6)	72.3 (68.9, 75.3)	57.0 (51.3, 62.1)
**Boosted**				
Overall	91.7 (90.6, 92.7)	95.1 (92.9, 96.6)	93.3 (92.1, 94.3)	90.6 (89.0, 91.9)
• AZD1222	95.2 (92.7, 96.8)	96.1 (93.3, 97.8)	96.2 (91.3, 98.3)	92.6 (82.7, 96.8)
• BNT162b2	91.8 (90.7, 92.8)	94.2 (90.8, 96.3)	93.2 (92.0, 94.2)	91.0 (89.4, 92.3)
• CoronaVac	88.8 (84.9, 91.7)	NA	NA	89.0 (85.2, 91.9)

Adjusted for age, age-squared, gender, state, nationality, and presence of comorbidities. Vaccine effectiveness for AZD1222 or BNT162b2 primary regimen taking CoronaVac as booster dose could not be calculated due to insufficient data. Abbreviations: VE, vaccine effectiveness; CI, confidence intervals; AZD1222, Oxford-AstraZeneca; BNT162b2, Pfizer-BioNTech; CoronaVac, Sinovac; NA, not available.

## Data Availability

The de-identified data shown in this research article are available upon request.

## Appendix A. Methodology—Screening Method

(A1)
$$VE_{Y,i}=\frac{PPV_{d}-PCV_{Y,d}}{PPV_{d}\times \left(1-PCV_{Y,d}\right)}$$

$$CI_{95\%}=1-\left(\left(1-VE_{Y,i}\right)\pm 1.96\times \sqrt{\frac{1-\frac{CV_{Y,d}}{V_{d}}}{CV_{Y,d}}+\frac{1-\frac{CnV_{Y}}{nV}}{CnV_{Y}}}\right)$$

This screening technique predicts the vaccine effectiveness (VE) against an outcome of a disease *Y* (total infection and specifically, symptomatic infection of COVID-19), for the vaccination status *d* (full or boosted vaccination, encompassing all types of vaccine), using the following variables calculated collectively. The variables are:

- PPV—The proportion of the population that is vaccinated against the disease (full or boosted vaccination, encompassing all types of vaccine), as of 31 March 2022.
- PCV—The proportion of outcomes (total infection and specifically, symptomatic infection) on the vaccinated population, from 1 January 2022 to 31 March 2022.
- CV—The number of outcomes that occurred within the vaccinated population, from 1 January 2022 to 31 March 2022.
- CnV—The number of outcomes that occurred within the unvaccinated population, from 1 January 2022 to 31 March 2022.
- V—The number of people in the total population who are vaccinated, as of 31 March 2022.
- nV—The number of people in the total population who are unvaccinated, as of 31 March 2022.

This method allows the comparison between observable PPV and PCV versus an unobserved counterfactual of vaccine effectiveness of 100%, or in other words, a PCV of zero. Despite its simplicity in terms of computation, the method does not allow the stratification by types of vaccines, due to the probable consequence of estimating VE inaccurately, as the PPV variable will be constrained to values less than one, in contrast to freely between zero (zero vaccine coverage) and one (full vaccine coverage).

## Appendix B. Age-Stratified Estimation of VE against COVID-19 Infections

**Table A1:** Variables in the calculation of VE using screening method.

Variables	Number of Individuals	
	COVID-19 Infection	Symptomatic COVID-19 Infection
**The number of outcomes that occurred within the vaccinated population, from 1 January 2022 to 31 March 2022, CV**		
• Vaccinated at least 2 doses	1,182,273	766,051
• Full vaccination	532,483	386,286
• Boosted vaccination	649,790	379,765
**The number of outcomes that occurred within the unvaccinated population, from 1 January 2022 to 31 March 2022, CnV**	235,947	156,333
**The number of people in the total population who are vaccinated, as of 31 March 2022, V**		
• Vaccinated at least 2 doses	25,805,722	
• Full vaccination	9,995,128	
• Boosted vaccination	15,810,594	
**The number of people in the total population who are unvaccinated, as of 31 March 2022, nV**	5,149,857	
**Estimate of current population of Malaysia**	32,657,100	

## Appendix C. Age-Stratified Estimation of VE against COVID-19 Infections

**Table A2:** Age-stratified estimation of VE against COVID-19 infections using screening method.

	VE (%), 95% CI				
	Age				
	<30	30–39	40–49	50–59	≥60
**COVID-19 Infection**					
Vaccinated at least two doses	96.3 (96.3, 96.3)	97.8 (97.8, 97.9)	98.9 (98.9, 98.9)	99.3 (99.2, 99.3)	99.2 (99.2, 99.3)
• Full Vaccination	93.6 (93.6, 93.6)	96.9 (96.9, 97.0)	98.6 (98.6, 98.6)	99.1 (99.1, 99.1)	99.0 (99.0, 99.0)
• Boosted Vaccination	98.9 (98.9, 98.9)	98.8 (98.7, 98.8)	99.2 (99.1, 99.2)	99.4 (99.4, 99.5)	99.5 (99.5, 99.5)
**Symptomatic COVID-19 Infection**					
Vaccinated at least two doses	97.5 (97.5, 97.5)	98.6 (98.5, 98.6)	99.3 (99.2, 99.3)	99.5 (99.5, 99.5)	99.5 (99.4, 99.5)
• Full Vaccination	95.6 (95.6, 95.7)	97.9 (97.8, 97.9)	99.0 (99.0, 99.0)	99.4 (99.3, 99.4)	99.3 (99.2, 99.3)
• Boosted Vaccination	99.3 (99.3, 99.3)	99.2 (99.2, 99.3)	99.5 (99.5, 99.5)	99.7 (99.6, 99.7)	99.7 (99.6, 99.7)

Abbreviations: VE, vaccine effectiveness; CI, confidence intervals.

## Appendix D. Baseline Characteristics of Study Cohort

**Table A3:** Baseline characteristics of the study cohort (*n* = 1,158,235) according to vaccination status.

Characteristic	Unvaccinated		Fully Vaccinated		Boosted		*p*-Value
	*n*	%	*n*	%	*n*	%	
**No. of Individuals**	51,988	4.5	480,495	41.5	625,752	54.0	
**Primary Regimen**							<0.001
AZD1222			52,089	10.8	84,515	13.5	
BNT162b2			297,881	62.0	251,309	40.2	
CoronaVac			130,525	27.2	289,928	46.3	
**Booster Dose**							
AZD1222					78,986	12.6	
BNT162b2					502,218	80.3	
CoronaVac					44,548	7.1	
**Death**							<0.001
Alive	50,922	97.9%	478,921	99.7%	625,185	99.9%	
Dead	1066	2.1%	1574	0.3%	567	0.1%	
**Gender**							<0.001
Female	22,782	43.8	259,518	54.0	329,803	52.7	
Male	29,206	56.2	220,977	46.0	295,949	47.3	
**Age Group**							<0.001
18 to 29	14,854	28.6	204,246	42.5	163,602	26.1	
30 to 39	13,589	26.1	132,721	27.6	180,799	28.9	
40 to 49	7942	15.3	60,881	12.7	121,602	19.4	
50 to 59	5155	9.9	38,911	8.1	82,334	13.2	
60 and above	10,448	20.1	43,736	9.1	77,415	12.4	
**Nationality**							<0.001
Non-Malaysian	15,199	29.2	10,164	2.1	14,356	2.3	
Malaysian	36,789	70.8	470,331	97.9	611,396	97.7	
**State**							<0.001
Johor	4798	9.2%	45,232	9.4%	52,297	8.4%	
Kedah	3902	7.5%	48,801	10.2%	45,087	7.2%	
Kelantan	2570	4.9%	36,575	7.6%	19,420	3.1%	
Melaka	1214	2.3%	12,763	2.7%	17,904	2.9%	
Negeri Sembilan	1884	3.6%	23,416	4.9%	35,034	5.6%	
Pahang	2413	4.6%	26,053	5.4%	25,385	4.1%	
Pulau Pinang	3270	6.3%	36,774	7.7%	48,303	7.7%	
Perak	2580	5.0%	20,727	4.3%	25,890	4.1%	
Perlis	358	0.7%	5226	1.1%	2244	0.4%	
Selangor	12,064	23.2%	115,520	24.0%	197,573	31.6%	
Terengganu	1566	3.0%	16,169	3.4%	13,867	2.2%	
Sabah	7080	13.6%	58,034	12.1%	36,867	5.9%	
Sarawak	2034	3.9%	5410	1.1%	30,146	4.8%	
W.P. Kuala Lumpur	5753	11.1%	24,664	5.1%	67,495	10.8%	
W.P. Labuan	370	0.7%	2475	0.5%	4720	0.8%	
W.P. Putrajaya	132	0.3%	2656	0.6%	3520	0.6%	
**Presence of Comorbidities**							<0.001
No Comorbidities	45,910	88.3%	429,441	89.4%	564,303	90.2%	
Comorbid	6078	11.7%	51,054	10.6%	61,449	9.8%	

Percentages for the number of individuals are of the total population, while percentages for each characteristic are within the respective vaccination status group. Abbreviations: BNT162b2, Pfizer-BioNTech; CoronaVac, Sinovac; AZD1222, Oxford-AstraZeneca.
